# Unveiling Boundary-Localized Interfacial Interactions in Temperature-Controlled Au-Assisted Exfoliation of MoS_2_ Monolayers

**DOI:** 10.3390/nano15231835

**Published:** 2025-12-04

**Authors:** Chaoqi Dai, Sikai Chen, Boyuan Wen, Bingrui Li, Lei Shao, Fangfei Ming, Shaozhi Deng

**Affiliations:** 1 State Key Laboratory of Optoelectronic Materials and Technologies, Guangdong Province Key Laboratory of Display Material and Technology, School of Electronics and Information Technology, Sun Yat-sen University, Guangzhou 510275, China; daichq5@mail2.sysu.edu.cn (C.D.); chensk5@alumni.sysu.edu.cn (S.C.); wenby6@mail2.sysu.edu.cn (B.W.); libr993@outlook.com (B.L.); shaolei5@mail.sysu.edu.cn (L.S.); 2 College of Physics and Electronic Engineering, Qilu Normal University, Jinan 250200, China

**Keywords:** Au-assisted exfoliation, MoS_2_, transition metal dichalcogenides, temperature-controlled interface, boundary effects

## Abstract

Gold-assisted exfoliation is an effective approach to obtain clean and large-area monolayers of transition metal dichalcogenides, yet the microscopic evolution of interfacial adhesion remains poorly understood. Here, we investigate temperature-controlled exfoliation of MoS_2_ between 30 and 170 °C. Based on optical microscopy image analysis, mild heating slightly improves the exfoliation yield, which is associated with the release of interfacial contaminants and trapped gases—these substances enhance the adhesion between gold and molybdenum disulfide (Au-MoS_2_). Unexpectedly, as revealed by AFM, SEM-EDS, and Raman analyses, parts of the Au film start to peel off from the underlying Ti adhesion layer at approximately 100 °C. This Au film detachment, resulting from the surprisingly weak Au-Ti adhesion, serves as a unique probe for interfacial strength: it preferentially occurs at the boundaries of MoS_2_ flakes, indicating that the reinforcement of the Au-MoS_2_ interaction originates at the edges rather than being uniformly distributed. At higher temperatures (>130 °C), Au detachment expands to larger areas, indicating that boundary-localized adhesion progressively extends across the entire interface. Additional STM/STS measurements further confirm that thermal annealing improves local Au-MoS_2_ contact by removing interfacial species and enabling surface reconstruction. These findings establish a microscopic picture of temperature-assisted exfoliation, highlighting the dual roles of interfacial contaminant release and boundary effects, and offering guidance for more reproducible fabrication of high-quality 2D monolayers.

## 1. Introduction

Transition metal dichalcogenides (TMDCs), such as MoS_2_, are layered van der Waals (vdW) semiconductors with remarkable electronic and optical properties, making them promising candidates for next-generation electronic and optoelectronic devices [1,2,3,4]. A key requirement for both fundamental studies and practical applications is the reliable fabrication of large-area, high-quality monolayers. Various approaches have been developed, among which the most widely used is mechanical exfoliation of bulk crystals onto substrates [5,6,7]. Contamination and trapped molecules at buried interfaces are known to strongly affect the exfoliation and transfer of two-dimensional (2D) materials. A common strategy to improve exfoliation yield and quality is moderate heating, which assists the release of interfacial residues and enhances vdW adhesion between contacting layers [8,9,10,11,12].

Gold-assisted exfoliation provides a particularly clean and efficient route for preparing monolayer TMDCs, with near-unity yields reported in many systems [13,14,15,16,17,18,19,20,21]. Its mechanism relies on the formation of strong Au–TMDCs interactions at the interface, while simultaneously weakening the interlayer interaction between the first and second MoS_2_ layers [22,23]. Previous studies have suggested that strong Au–MoS_2_ bonding sites are sparse and randomly distributed across the interface [24]. In practice, successful exfoliation of monolayers depends on a delicate balance of adhesion energies among multiple competing interfaces. This method has been demonstrated in a broad range of metal–2D material systems, establishing a robust route toward cost-effective and uniform wafer-scale monolayer production [25,26,27,28]. Beyond large-area exfoliation, metal-assisted methods have also been explored for fabricating functional structures, such as stacking layers [29,30], twisted heterostructures [31], patterns [26,32] and device arrays [32]. However, it is still challenging to fabricating TMDC patterns high accuracy, typically to the micrometer scale, since smaller Au contact regions generally fail to sustain stable exfoliation of TMDCs [32,33,34,35,36]. A clearer microscopic picture of where strong Au–MoS_2_ connections first form, and how they evolve under processing conditions, would therefore not only deepen our understanding of the exfoliation mechanism, but also provide useful guidance for realizing complex patterned 2D structures using metal-assisted exfoliation.

In this work, we investigate Au-assisted exfoliation of MoS_2_ under controlled substrate temperatures ranging from 30 to 170 °C. Because the interface in this exfoliation system is confined between two solid layers and undergoes continuous transformation during heating, conventional high-resolution probes cannot directly resolve its structure or chemistry. Thus, the interfacial interactions are best inferred from the exfoliated product morphology and adhesion contrast, for which optical microscopy provides the most comprehensive and scalable characterization. Mild heating promotes the release of interfacial contaminants, thereby strengthening the Au–MoS_2_ interaction and slightly improving the exfoliation yield. Unexpectedly, the Au film itself can detach from the underlying Ti adhesion layer due to weak Au–Ti bonding. Although this detachment is not intrinsic to MoS_2_ exfoliation, it provides a unique probe for mapping the spatial distribution of the Au–MoS_2_ adhesion. By tracking this effect, we identify that reinforcement of the strong vdW Au–MoS_2_ interaction originates at the flake boundaries and progressively extends across the entire interface with increasing temperature. This finding establishes a new perspective on the formation of strong vdW metal–2D semiconductor contacts and underscores the critical role of domain boundaries in determining exfoliation pathways.

## 2. Materials and Methods

10 × 10 mm^2^ Si substrates with a ~300 nm SiO_2_ layer were used as the substrates. A ~2 nm Ti adhesive layer and an ~18 nm Au film were sequentially deposited by magnetron sputtering. Bulk MoS_2_ crystals (Taizhou SUNANO New Energy, Shanghai, China) were mechanically exfoliated using Kapton polyimide tape, which offers thermal stability up to ~300 °C. To provide comparable mechanical support to conventional blue tape, five layers of Kapton tape were stacked during exfoliation. The tape was gently covered onto the Au-coated substrate after the Au film was exposed to air for ~1 min, minimizing pressure effects to avoid damaging the material. The samples were heated on a hot plate at the designated substrate temperature for 10 min before tape removal. The temperature was monitored in real time using a TES-1310 digital thermometer (TES, Taipei, Taiwan, China) with a K-type thermocouple placed in direct contact with the sample to ensure accurate readings of the actual sample temperature. For post-exfoliation thermal treatments, selected MoS_2_/Au/Ti/SiO_2_/Si samples were annealed in a quartz-tube furnace under Ar flow (30 sccm, 20 min).

Optical microscopy (Zeiss LSM700 and AxioScope AI, Oberkochen, Germany) was used to characterize exfoliated MoS_2_ domains. The coverage of various surface structures in optical microscope images was determined from three representative 1 × 1 mm^2^ regions with the highest MoS_2_ coverage on each sample, using Fiji/ImageJ (version 1.54f) for area selection. Raman spectra were collected with a RENISHAW inVia system (London, UK) using a 532 nm laser at 0.6 mW. AFM images were acquired with a Bruker Dimension Fastscan (Billerica, MA, USA) in peak force tapping mode under ambient conditions.

Scanning tunneling microscopy (STM) measurements are carried out using a variable temperature STM (Omicron, Taunusstein, Germany) operating in ultra-high vacuum (base pressure better than 1 × 10^−9^ mbar) at room temperature. Point d*I*/d*V* spectra are obtained by differentiating the smoothed *I*(*V*) curves.

## 3. Results and Discussion

**Figure 1:**
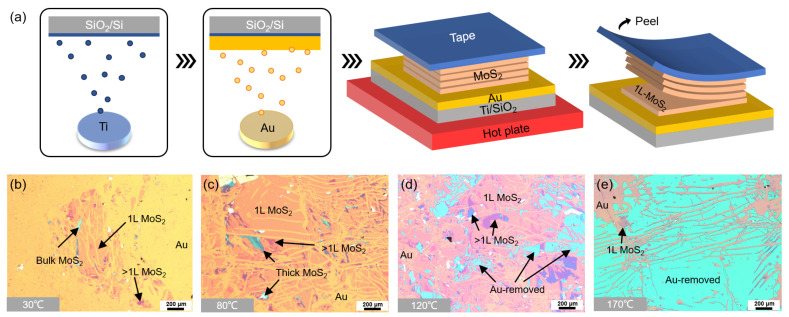
**Au-assisted exfoliation of MoS_2_ monolayers at different substrate temperatures.** (**a**) Schematic illustration of the exfoliation procedure. (**b**–**e**) Optical microscope images of exfoliated samples prepared at 30 °C, 80 °C, 120 °C, and 170 °C, respectively. Regions of exposed Au, monolayer MoS_2_, multilayer MoS_2_, bulk residues, and Au-removed areas are labeled.

As illustrated in Figure 1a, to examine the role of substrate temperature in Au-assisted exfoliation, we first deposited a Ti adhesion layer (~2 nm) on Si/SiO_2_ substrates by magnetron sputtering, followed by Au thin films (~18 nm). Due to the limitations of our deposition system, the samples had to be exposed to air for less than 1 min after Ti deposition before being reloaded into vacuum for Au deposition. This unavoidable air exposure at the Ti–Au interface is later found to be closely related to the Au-removal phenomena discussed below.

After preparing the Au films, bulk MoS_2_ crystals attached to adhesive tape were gently placed onto the Au surface without pressing, in order to minimize possible pressure effects. The samples were then heated on a hot plate at the target temperature for 10 min. Upon peeling off the tape, part of the top MoS_2_ monolayer remained on the Au due to the strong vdW interaction between MoS_2_ and Au. Optical microscope images (Figure 1b–e) reveal the overall temperature dependence of the exfoliation results. At 30 °C (Figure 1b), only ~30% of the Au surface was covered by monolayer MoS_2_, indicating insufficient interfacial adhesion. At 80 °C (Figure 1c), the exfoliation yield increased dramatically, with most of the surface covered by monolayer MoS_2_, although multilayer flakes and bulk residues were still present. At 120 °C (Figure 1d), in addition to the MoS_2_ regions, uniformly blue-colored areas appeared, which differed from the optical contrast of monolayers or multilayers and were identified as Au-removed regions. At 170 °C (Figure 1e), exfoliation of MoS_2_ was almost completely suppressed, and more than 60% of the surface consisted of Au-removed regions.

To quantify the temperature dependence of exfoliation, we carried out systematic experiments from 30 to 170 °C at ~10 °C intervals. The core rationale for selecting 170 °C as the upper limit is that the adhesive tape undergoes denaturation at temperatures above this threshold. Additional optical images are summarized in Figure S1. Following the statistical method in our previous paper (Ref. [23]), three parameters were defined: the total exfoliation yield of MoS_2_ [$Y=\sum_{k=1}^{3}(S_{all}^{k}/S_{fov}^{k})/3\times 100\%$], the area fraction of monolayer regions within all MoS_2_ covered regions [$F_{1L}=\sum_{k=1}^{3}(S_{1L}^{k}/S_{all}^{k})/3\times 100\%$], and the area fraction of Au-removed regions [$F_{\mathrm{Au\_r}}=\sum_{k=1}^{3}(S_{\mathrm{Au\_r}}^{k}/S_{fov}^{k})/3\times 100\%$], where *S_fov_* is the area of each selected optical microscope image, *S_all_* is the total area covered by MoS_2_, *S_1L_* is the area covered by monolayer MoS_2_, and *S_Au_r_* is the area of Au-removed regions. The results reported here are based on three representative 1 mm × 1 mm images with the highest MoS_2_ coverage on each sample (see Ref. [24]).

**Figure 2:**
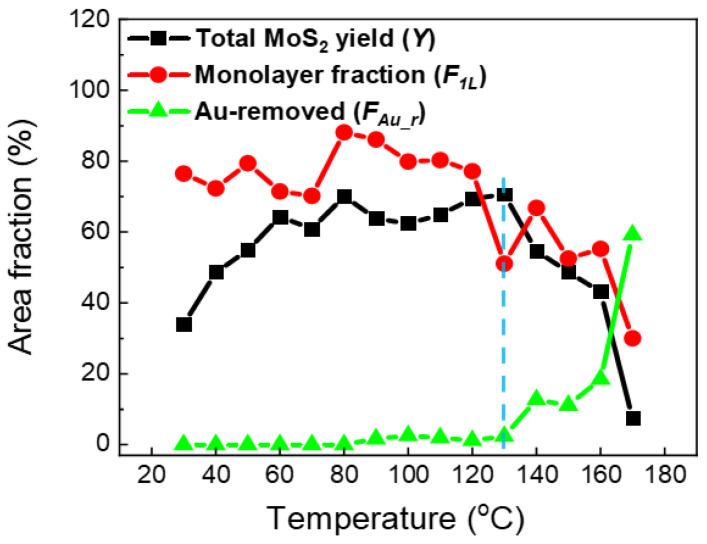
**Temperature dependence of exfoliation yield and area fractions.** Statistical analysis of optical images gives the total MoS_2_ yield (*Y*), the monolayer fraction (*F*_1_*_L_*), and the fraction of Au-removed regions (*F_Au_r_*). The dashed vertical line at T = 130 °C marks the onset of Au removal and the simultaneous drop in MoS_2_ yield.

As shown in Figure 2, the total yield *Y* increased from ~30% at 30 °C to ~70% at 80 °C, and remained nearly constant (~70%) up to 130 °C. The Au–MoS_2_ interface involves chemically inert surfaces, and the strengthening of their interaction upon heating arises primarily from physical effects. The gradual increase in yield between 30 and 80 °C can be attributed to the release of interfacial contaminants and trapped gas molecules, which enhances the effective Au–MoS_2_ adhesion and facilitates more efficient exfoliation. This temperature range is consistent with previous reports on the effective release of interfacial gas bubbles near 110 °C [12]. Above 130 °C, however, *Y* dropped sharply, reaching ~5% at 170 °C. In contrast, $F_{\mathrm{Au\_r}}$ was negligible below 80 °C, slightly increased to ~3% between 90 and 130 °C, and then rose steeply above 130 °C, reaching ~60% at 170 °C. The monolayer fraction *F_1L_* was consistently high (~80%) below 130 °C, but decreased significantly at higher temperatures, dropping to ~30% at 170 °C. These results clearly indicate that 130 °C represents a threshold temperature: above this point, MoS_2_ exfoliation is strongly suppressed, and large Au-removed regions begin to dominate.

**Figure 3:**
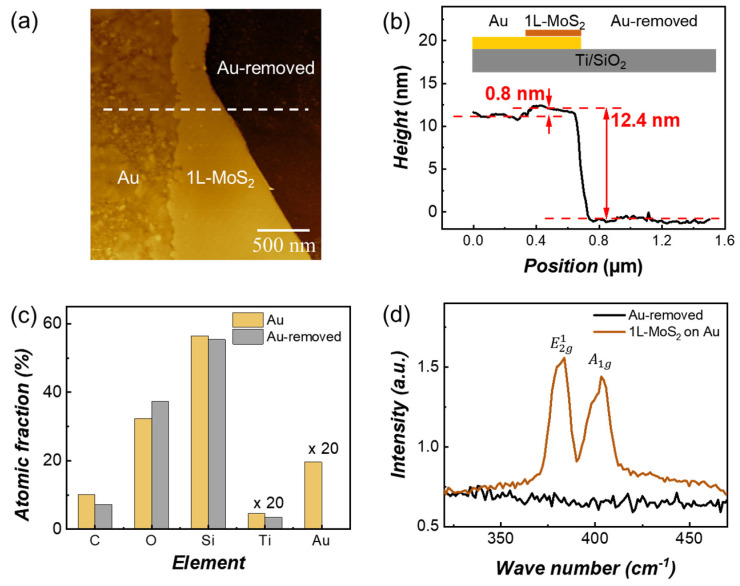
**Characterization of the Au-removed regions.** (**a**) AFM image showing the boundary between a bare Au area, a 1L-MoS_2_-covered area, and an Au-removed area. (**b**) AFM line profile extracted along the dashed line in (**a**). (**c**) Atomic fractions of Au-covered and Au-removed regions obtained from SEM-EDS analysis (×20 indicates a 20-fold magnification of the atomic fraction in the corresponding plot). (**d**) Raman spectra of 1L-MoS_2_ and Au-removed regions.

To further clarify the composition and characteristics of the Au-removed regions, we performed complementary analyses. Figure 3a shows an AFM image containing three distinct regions: bare Au, 1L-MoS_2_, and an Au-removed area. The corresponding line profile extracted along the dashed line in Figure 3a is presented in Figure 3b. The step height between the bare Au and the 1L-MoS_2_ region is ~0.8 nm, consistent with the expected thickness of a monolayer. In contrast, the Au-removed region exhibits a much larger height drop of ~12.4 nm relative to the 1L-MoS_2_, indicating the absence of the Au film in this region.

Elemental analysis by SEM-EDS (Figure 3c, the corresponding SEM image is shown in Figure S2) further confirms this observation. In the Au-covered region, the atomic fraction of Au is ~1%, whereas in the Au-removed region it is nearly zero. The other elements (C, O, Si, and Ti) show only minor variations between the two regions, suggesting that the primary difference arises from the loss of the Au film. Raman spectroscopy (Figure 3d) provides additional evidence. In the 1L-MoS_2_ region, the characteristic $E_{2g}^{1}$ (~383 cm^−1^) and $A_{1g}$ (~403 cm^−1^) vibrational modes of MoS_2_ are clearly observed. By contrast, these peaks are absent in the Au-removed region, indicating that no MoS_2_ monolayer remains there. Together, these results confirm that the Au-removed regions correspond to areas where the Au film has detached along with the MoS_2_ attached to the tape.

The removal of the Au layer from the Ti adhesion layer is unexpected, as the Ti–Au interface is expected to be stabilized by ionic or metallic bonding, which should be much stronger than any vdW-type interfaces. Room-temperature control tests further show that Kapton tape alone cannot peel off Au films without MoS_2_ flakes, indicating decently strong Au/Ti adhesion without heating. The weakening of this boundary is likely related to air exposure of the Ti surface prior to Au deposition, which may lead to oxidation or contamination of the Ti layer. To verify this, we deliberately extended the air exposure of the Ti surface to 10 min before Au deposition. The resulting samples exhibited a much less stable Au/Ti interface, where Au could be removed even during room-temperature exfoliation, as shown in Figure S3. This result confirms that the adhesion of the Au/Ti interface is highly sensitive to surface contamination and can act as the weak link in exfoliation under certain conditions. The weak adhesion of the Au film is consistent with previous reports showing that Au films can be detached by adhesive tapes due to weak Au–oxide bonding [37,38].

**Figure 4:**
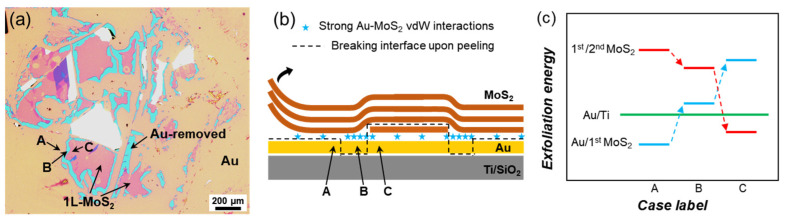
**Formation mechanism of Au-removed regions.** (**a**) Optical micrograph of a sample prepared at 120 °C, where Au removal first emerges. Narrow Au-removed regions are seen surrounding 1L-MoS_2_ domains, indicating their correlation with MoS_2_ boundaries. (**b**) Schematic illustration of the local interfacial configuration, showing three adjacent regions: A, B, and C, corresponding to bare Au, Au-removed, and 1L-MoS_2_-covered regions, respectively. The higher density of star symbols near region B indicates the locally enhanced strong vdW interactions between Au and MoS_2_ near the flake boundaries. (**c**) Schematic comparison of exfoliation energies for different interfaces in the three regions, taking the Au/Ti interface as reference.

Since the Au/Ti interface lies beneath the Au/MoS_2_ interface, its adhesion is expected to be relatively uniform across the substrate. However, the spatial distribution of Au-removed regions is not random. As seen in Figure 1d,e, the morphology of the Au-removed patches appears very similar to the geometry of 1L-MoS_2_ domains. This correlation is most clearly observed when Au removal first appears. As shown in Figure 4a, narrow Au-removed regions surround isolated MoS_2_ flakes, implying that Au detachment preferentially initiates along MoS_2_ domain boundaries. This observation rules out the possibility that Au/Ti rupture dictates MoS_2_ coverage. Instead, it indicates that the pre-existing structural boundaries of MoS_2_ act as templates that guide where Au removal nucleates at around 100 °C. It should be noted that, because the Kapton tape had been repeatedly used to cleave bulk MoS_2_, its surface became more than 90% covered by MoS_2_ flakes, making direct tape–Au contact marginal during exfoliation. The remaining exposed regions cannot account for the Au film removal, as the Au-detached areas follow the geometry of the exfoliated MoS_2_ flakes (Figure 4a). Even if direct tape–Au adhesion had increased at elevated temperatures, it would not affect our interpretation, as the observed Au detachment is spatially locked to the MoS_2_ edge-exfoliation boundaries rather than to the tape–Au contact area.

To explain this behavior, we propose a schematic model, as shown in Figure 4b. The bonding forces between metals as well as between metals and semiconductors have been thoroughly studied [39,40,41]. A finite MoS_2_ flake embedded between the Au film and upper MoS_2_ layers introduces geometric boundaries. At these boundaries, the Au–MoS_2_ interaction becomes locally enhanced, illustrated by the higher density of star markers. This configuration produces three adjacent interfacial regions: labelled as A, B, and C as seen in the image. The corresponding exfoliation energies are compared in Figure 4c. In region A, exfoliation occurs at the Au-MoS_2_ interface. Although strong vdW interactions may randomly form at the Au–MoS_2_ interface, exfoliating the first MoS_2_ layer requires generating a tear boundary in its covalent network (i.e., breaking local covalent bonds at the edge, as shown in Ref. [42]), which leads to a relatively high exfoliation energy at the 1st/2nd MoS_2_ interface. In region C, exfoliation takes place at the 1st/2nd MoS_2_ interface, where only the weaker interlayer vdW force is to overcome, making it energetically favorable. In region B, the locally enhanced Au–MoS_2_ interaction increases the Au/1L-MoS_2_ interfacial energy above that of the Au/Ti interface (while simultaneously decreasing the 1st/2nd MoS_2_ binding energy slightly, see Ref. [22]). Thermal expansion mismatch between oxidized Ti and Au may also contribute to the reduced adhesion and subsequent detachment of the Au layer [43,44]. As a result, exfoliation preferentially occurs at the Au/Ti interface, leading to Au detachment.

The origin of the enhanced Au–MoS_2_ adhesion at flake boundaries may involve multiple effects. One possibility is the faster release of trapped interfacial gas molecules, possibly facilitated by diffusion pathways at these MoS_2_ flake boundaries, therefore improving the interfacial contact. Another is enhanced Au diffusion along MoS_2_ boundaries, consistent with earlier reports, see Ref. [42], which increases the effective contact area and strengthens adhesion. These possibilities are in line with previous atomistic ab initio studies showing that interfacial bonding energies in metal–semiconductor and vdW heterostructures are highly sensitive to the local chemical environment and contact configuration [39,40,41].

**Figure 5:**
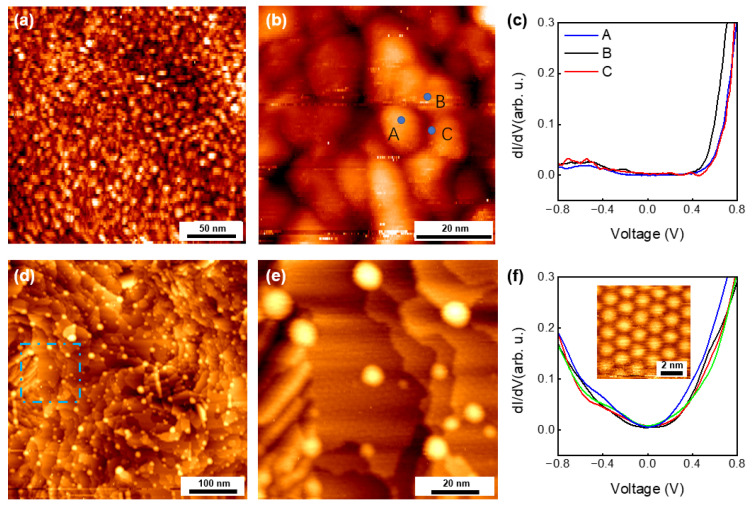
**STM characterization of the MoS_2_/Au interface before and after annealing.** (**a**) STM overview image of the as prepared MoS_2_/Au interface (*V*_S_ = −2 V, *I*_t_ = 0.05 nA). (**b**) Enlarged STM image in a region in (**a**) (*V*_S_ = −1 V, *I*_t_ = 0.1 nA). (**c**) dI/dV spectra collected at three sites marked in (**b**). (**d**) STM overview image of the 350 °C annealed MoS_2_/Au interface (*V*_S_ = −2 V, *I*_t_ = 0.05 nA). (**e**) Enlarged STM image in regions marked in darshed blue box in (**d**) (*V*_S_ = −1 V, *I*_t_ = 0.1 nA). (**f**) dI/dV spectra collected at four sites in flat terraces. The inset STM image shows the moiré superlattices formed due to the lattice mismatch between MoS_2_ and Au(111) on flat terrace regions.

To further probe the evolution of the buried MoS_2_/Au interface, we performed STM and STS measurements on samples before and after thermal annealing, as shown in Figure 5. The as-prepared interface exhibits a highly corrugated and amorphous-like morphology (Figure 5a,b). The dI/dV spectra collected from these regions display semiconducting behavior (Figure 5c), indicating weak electronic hybridization and limited formation Au–MoS_2_ contact sites. This weak contact suggests the presence of trapped gases and contaminants between MoS_2_ and Au.

After annealing at 350 °C, the interface undergoes a pronounced reconstruction. Large, atomically flat terraces emerge as gold atoms reorganize following the release of interfacial contaminants (Figure 5d,e). Within these terraces, well-defined moiré superlattices characteristic of the MoS_2_/Au(111) heterointerface become visible, demonstrating significantly improved contact quality. Correspondingly, the STS spectra acquired on these terraces exhibit metallic features (Figure 5f), consistent with previous reports attributing such metallic behavior to strong interfacial hybridization between MoS_2_ and Au [42]. This enhanced hybridization only occurs when MoS_2_ is brought into intimate contact with a re-constructed Au surface.

These STM/STS results demonstrate that thermal annealing plays a crucial role in improving the microscopic contact of the MoS_2_/Au interface. Heating removes trapped interfacial species and allows surface Au atoms to reconstruct into flat terraces, which in turn enables MoS_2_ to form much more intimate contact with the Au(111) surface. Although STM does not directly resolve the buried interface, these observations suggest that regions where contaminants are removed more rapidly can more readily develop stronger local adhesion sites. Considering that molecular or atomic diffusion is expected to be faster near the porous edge structure of MoS_2_, it is reasonable to infer that such processes may initiate more efficiently at the flake boundaries, consistent with the boundary-related behavior observed in our morphology-based analysis.

**Figure 6:**
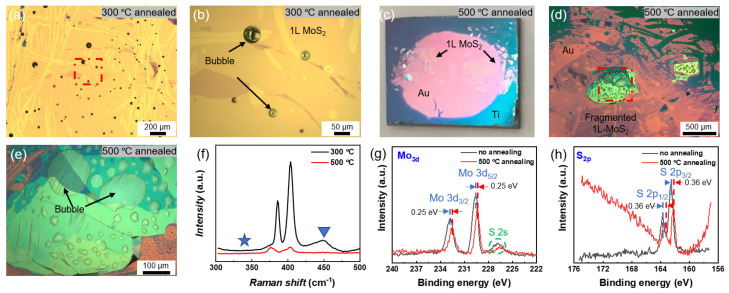
**Effects of high-temperature annealing on 1L-MoS_2_/Au samples.** Samples were annealed in a tube furnace under 30 sccm Ar flow for 20 min. (**a**) Optical micrograph after annealing at 300 °C. (**b**) Magnified view of the boxed region in (**a**), showing several Au film bubbles unrelated to MoS_2_ boundaries. (**c**) Optical image of a 1 cm^2^ Si wafer slice after annealing at 500 °C, showing disappearance of Au near the wafer edge and blue contrast from exposed Ti due to lateral Au diffusion. (**d**) Optical micrograph of a region where the Au film remains after 500 °C annealing. Some 1L-MoS_2_ domains are still recognizable though fragmented. (**e**) Magnified view of the boxed region in (**d**), showing a thick MoS_2_ region that remains intact except for several trapped bubbles between MoS_2_ and Au. (**f**) Raman spectra of 1L-MoS_2_ regions after annealing at different temperatures, showing clear degradation of MoS_2_ after 500 °C treatment (The pentagrams and triangles at ~335 cm^−1^ and ~454 cm^−1^ indicate the characteristic peaks (see text)). XPS spectra of samples without annealing and after 500 °C annealing: (**g**) Mo 3d; (**h**) S 2p.

At higher temperatures (e.g., 170 °C), Au removal extends across the entire surface. This behavior may result from further releasing of interfacial contaminations, or from Au diffusion into MoS_2_ interlayers, which increases the binding energy between the first and second MoS_2_ layers. The possible reduction in Au/Ti adhesion energy due to interfacial reactions (such as trapped gas expansion or Ti oxidation) might also play a role. To further probe these possibilities, we tested the thermal stability of exfoliated samples at elevated temperatures. As shown in Figure 6a,b, annealing the sample at 300 °C produces localized Au bubbles uncorrelated with MoS_2_ boundaries, consistent with trapped gas effects at the Au/Ti interface. At 500 °C, as shown in Figure 6c–e, large-scale Au diffusion occurs, with Au migrating laterally away from the wafer edge and MoS_2_ domains becoming fragmented. No Raman signatures of phase or chemical-state modification are observed at 300 °C (Figure 6f), the temperature range relevant to the present exfoliation study. Raman spectra further reveal severe degradation of 1L-MoS_2_ after 500 °C annealing.

Such extreme high-temperature annealing (500 °C) induces detectable changes in the local chemical state of MoS_2_. Figure 6g displays the XPS spectra of the Mo 3d and S 2s orbitals in MoS_2_ before and after annealing. For the unannealed sample (black curve), the Mo 3d orbital splits into characteristic peaks of 3d_5/2_ (~229.6 eV) and 3d_3/2_ (~232.8 eV). After 500 °C annealing (red curve), both characteristic peaks of Mo 3d shift 0.25 eV toward lower binding energies. Additionally, the peak appearing on the low binding energy side of the Mo 3p band (~226.9 eV) corresponds to the S 2s orbital signal, a typical XPS feature of S 2s in MoS_2_. Figure 6h presents the S 2p orbital in MoS_2_ before and after annealing. After 500 °C annealing, both characteristic peaks of S 2p shift 0.36 eV toward lower binding energies, consistent with the shift trend of the Mo 3d peaks in Figure 6g. Together, these results suggest that both interfacial gas and Au diffusion might play important roles in the formation and evolution of Au-removed regions. In addition, although high temperature treatment had been shown to create coexisting 1T phase for MoS_2_ [45,46], our Raman data does not indicate a clear signature (no peak at 335 cm^−1^, as shown in Figure 3d and Figure 6) [47].

## 4. Conclusions

In summary, we have systematically examined temperature-controlled Au-assisted exfoliation of MoS_2_ in the range of 30–170 °C. At low temperatures (30–80 °C), the exfoliation yield increases modestly, which we attribute to the gradual release of interfacial contaminants and trapped gas molecules that enhances the effective Au–MoS_2_ adhesion. At intermediate temperatures (~80–120 °C), Au-removed regions emerge and nucleate preferentially along the edges of MoS_2_ flakes. Although this Au removal originates from rupture at the buried Au/Ti interface, it serves as a unique probe for locating where interfacial adhesion is strongest, revealing that the strengthening of the Au–MoS_2_ interaction initiates at domain boundaries. At higher temperatures (>130 °C), widespread Au removal dominates, indicating that the boundary-localized adhesion enhancement progressively extends across the entire interface, likely due to further expulsion of interfacial gases and enhanced Au diffusion. Complementary STM/STS measurements further confirm that thermal annealing promotes the removal of interfacial species and the reconstruction of surface Au into flat terraces, thereby enabling more intimate and electronically coupled Au–MoS_2_ contact. Taken together, these results clarify the microscopic processes underlying temperature-assisted exfoliation and highlight the critical role of domain boundaries in governing interfacial interactions, offering new insights for designing more reliable strategies to fabricate large-area 2D semiconductor monolayers.

## Data Availability

The data are contained within the article and Supplementary Materials.

## Supplementary Materials

The following supporting information can be downloaded at: https://www.mdpi.com/article/10.3390/nano15231835/s1, Figure S1: Additional optical microscope images of samples prepared via temperature-controlled Au-assisted exfoliation. Figure S2: SEM images of a sample surface region with neighboring bare Au area and the Au-removed area. Figure S3: Optical microscopy image of a sample prepared at 30 °C using a Au film deposited after prolonged air exposure of the Ti surface.
