# Supplementary material for: Unveiling Boundary-Localized Interfacial Interactions in Temperature-Controlled Au-Assisted Exfoliation of MoS2 Monolayers
Source: Nanomaterials (Basel). 2025 Dec 4;15(23):1835. doi: 10.3390/nano15231835 (PMC12693429; doi:10.3390/nano15231835)
Supplement: Supplementary file 1 [file nanomaterials-15-01835-s001.zip › nanomaterials-3991637-supplementary.pdf]

Supplementary materials

Unveiling Boundary-Localized Interfacial Interactions in Temperature-Controlled Au-Assisted Exfoliation of MoS_2 Monolayers

Chaoqi Dai [1,†], Sikai Chen [1,†], Boyuan Wen [1], Bingrui Li [1,2], Lei Shao [1], Fangfei Ming [1,*] and Shaozhi Deng [1,*]

1 State Key Laboratory of Optoelectronic Materials and Technologies, Guangdong Province Key Laboratory of Display Material and Technology, School of Electronics and Information Technology, Sun Yat-sen University, Guangzhou 510275, China;
daichq5@mail2.sysu.edu.cn (C.D.); chensk5@alumni.sysu.edu.cn (S.C.);
wenby6@mail2.sysu.edu.cn (B.W.);
libr993@outlook.com (B.L.); shaolei5@mail.sysu.edu.cn (L.S.)
2 College of Physics and Electronic Engineering, Qilu Normal University,
Jinan 250200, China
* Correspondence: mingff@mail.sysu.edu.cn (F.M.); stsdsz@mail.sysu.edu.cn (S.D.)
† These authors contributed equally to this work.

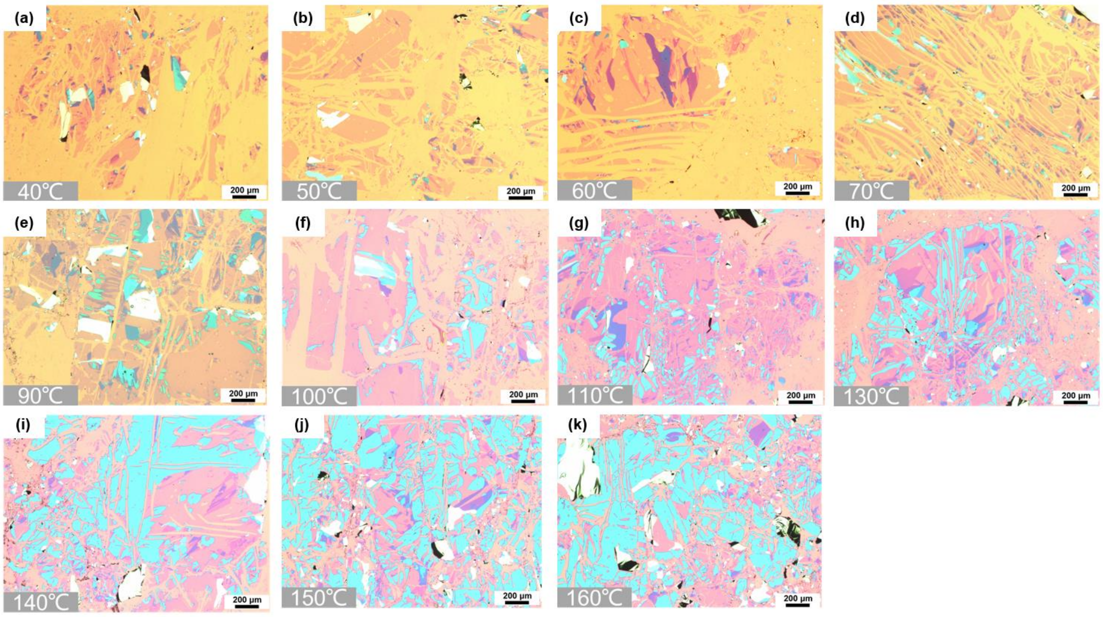

Figure S1. Additional optical microscope images of samples prepared via temperature-controlled Au-assisted exfoliation. In panels (a–d), the blue patches mainly correspond to multilayer MoS_2 regions, whereas in panels (e–k), blue areas predominantly correspond to Au-removed regions.

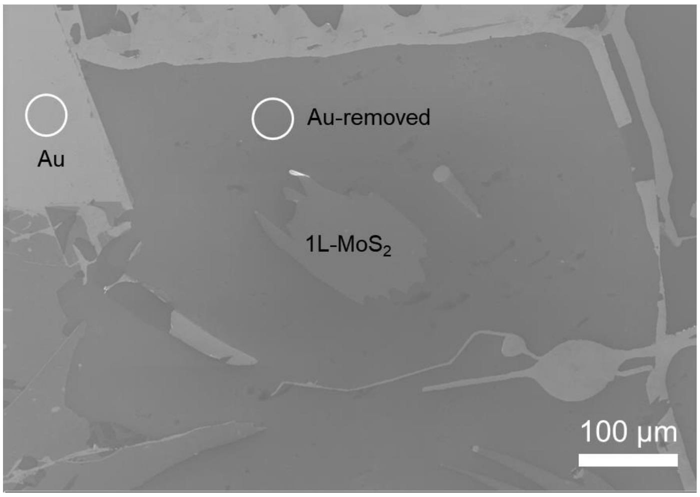

Figure S2. SEM images of a sample surface region with neighboring bare Au area and the Au-removed area. The circles indicate the positions for SEM-EDS measurements.

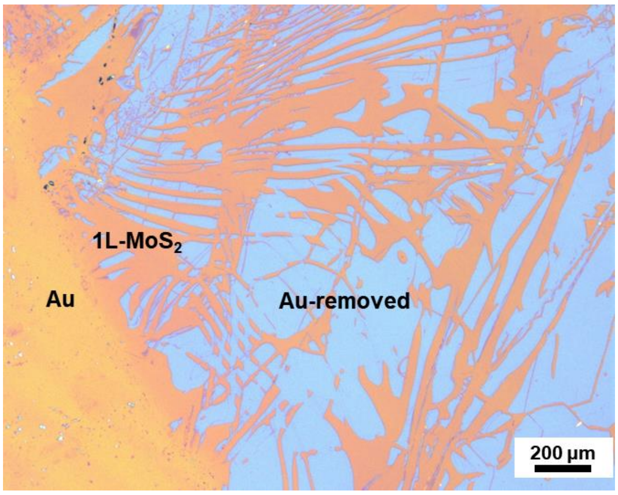

Figure S3. Optical microscopy image of a sample prepared at 30 °C using an Au film deposited after prolonged air exposure of the Ti surface. The purple regions correspond to Au-removed areas, in contrast to the blue regions in other optical images shown in this work. The color difference arises from using a different optical microscope and light source.
